# Spontaneous formation and optical manipulation of a woven domain fabric in a ferroelectric crystal

**DOI:** 10.1038/s41377-026-02374-7

**Published:** 2026-07-14

**Authors:** Feifei Xin, Yehonatan Gelkop, Ewout van der Veer, Beatriz Noheda, Ludovica Falsi, Guoquan Zhang, Fang Bo, Aharon J. Agranat, Eugenio DelRe

**Affiliations:** 1 The MOE Key Laboratory of Weak-Light Nonlinear Photonics, School of Physics and TEDA Applied Physics Institute, Nankai University, Tianjin, 300071 China; 2 Dipartimento di Fisica, Università di Roma “La Sapienza”, Rome, 00185 Italy; 3 The Institute of Applied Physics, The Hebrew University, Jerusalem, 91904 Israel; 4 Zernike Institute for Advanced Materials, University of Groningen, Groningen, 9747AG The Netherlands; 5 Groningen Cognitive Systems and Materials Center (CogniGron), University of Groningen, Groningen, 9747AG The Netherlands

**Keywords:** Nonlinear optics, Optical materials and structures

## Abstract

Advances in ferroelectric materials, including engineered thin films and solid solutions, have enabled reliable photonic memory devices using switchable polarization states, providing fast, non-volatile, and energy-efficient optical data storage. Moreover, the ability to optically manipulate these encoded domain structures allows precise, reconfigurable light control, further enhancing photonic memory performance and versatility. We report the first observation of a robust, woven fabric of interlaced domains that spontaneously forms in bulk KTN:Li as it undergoes the ferroelectric phase transition, and demonstrate its local manipulation using focused light. The braided domain structure emerges as an extended irregular topologically-protected defect with an embedded distribution of locked-in charged domain walls, activation points that allow site-by-site manipulation using visible laser. Our discovery of woven fabric that can be optically addressed introduces a new route to achieve extended solid-state topologically-protected photonic memory.

## Introduction

Ferroelectric materials can store information in their spontaneous polarization distribution, forming the basis for highly efficient electronic and photonic memory storage devices^1,2^. Optically-driven polarization enables read, write, and erase memory operations, allowing precise and reconfigurable light-based control^3,4^. Beyond conventional storage in simple polarization domains, topologically protected dipolar textures, such as vortices, skyrmions, and merons^5–9^, can provide inherently robust and noise-resistant information storage. In functional systems, such as ferromagnetic and ferroelectric materials, one fundamental mechanism that generates topologically protected structures is spontaneous symmetry breaking^10^. Given the finite extension of a system undergoing spontaneous symmetry breaking, lower symmetry domains nucleate independently, grow, and ultimately interact, forming topologically stable structures known as topological defects or topological solitons^11,12^. Topological protected structures also occur in complex biological systems, where information is securely stored in extended woven structures, such as the double-helical structure of DNA and the bundled fiber networks of the brain^13,14^. The key to enhanced stability and plasticity lies in the inherent structural geometry, whereby each composing thread extends and weaves throughout the entire fabric, so that short of cutting the threads, the fabric will maintain its integrity irrespective of external perturbations or noise. While previously reported topological defects, such as vortices and skyrmions, can solidify into extended superlattices with remarkable properties^15–21^, the resulting structure is a tesselation of localized domains. Weaving involves a complex and coordinated 3D threading that has never been observed to emerge spontaneously in a solid-state ferroelectric crystal out of symmetry breaking, and biomimetic electrical and photonic structural memory based on interlaced domains has not been previously studied.

We here report the spontaneous formation and optical manipulation of an irregular ferroelectric domain fabric in a bulk crystal below its room-temperature Curie point. The phenomenon is found in potassium lithium tantalate niobate (KTN:Li), a solid-solution perovskite that hosts a rich variety of topological defects with remarkable optical properties, including three-dimensional supercrystals that enable broadband giant refraction and constraint-free wavelength conversion^15,16^. In our experiments, domain fabric self-morphs out of a superlattice 2 K below the Curie point in proximity of the crystal surface. Weaving is observed to be irregular and history-dependent, i.e., the interlacing varies randomly for different thread-crossing points, a complex 3D robust patterning that we are able to alter and manipulate using focused visible laser light. While current efforts focus on optical manipulation of isolated topological defects, control of a woven domain network, with locked-in crossings, opens a route towards stable and energy-efficient photonic information platforms for neuromorphic computing and artificial intelligence^22^.

## Results

### Spontaneous woven domain fabric

**Fig. 1:**
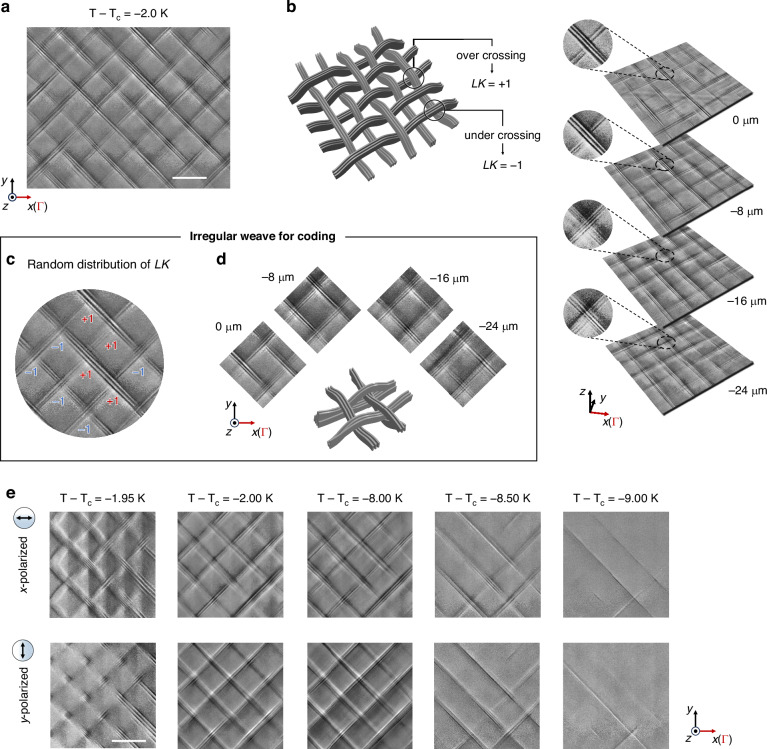
Spontaneous interwoven ferroelectric domain fabric. **a** Microscope imaging of a woven domain network. **b** Evidence of spontaneous ferroelectric domain weaving using multifocus imaging. Examples of images taken at various depths (Δ*z* = 0, −8, −16, −24 μm) are presented in the right panel with a corresponding 3D illustration (left inset). **c** 3D woven fabric characterized by an irregular distribution *LK*s. **d** Mesh complexity observed using multifocus imaging (top) and the corresponding illustration (bottom). **e** Microscope images of the temperature-driven spontaneous formation of the woven domain fabric using x- (top row) and y-polarized (bottom row) light. The sample is zero-cut with the growth direction Γ along x. Scale bar is 10 μm

Evidence of ferroelectric domain weaving in a transparent KTN:Li crystal using white-light optical microscopy is reported in Fig. 1 (see “Method” section). The sample dimensions are 3.4 mm (*x*) × 2.1 mm (*y*) × 0.52 mm (*z*), with the crystal growth direction Γ along the *x* axis (see Fig. S1a). The sample has a periodic striation pattern of composition caused by segregation during growth that characterize bulk crystals^23^. This compositional modulation produces a spatial variation of the Curie temperature *T*_*C*_(*x*), with the average Curie point being *T*_*C*_ = 292 K (see Fig. S1b)^24^. As the sample is cooled below the Curie point into the tetragonal phase, spontaneous polarization forms along the principal axes (see Fig. S3b). Figure 1a displays the microscope image of a part of the woven domain network observed at *T* − *T*_*C*_ = −2.0 K. The domain fabric emerges from the built-in periodic striation pattern associated to the bulk crystal growth, with a lattice constant of 10 *μ*m equal to twice the striation period. The weaving remains robust over the temperature interval −2.0 K > *T* − *T*_*C*_ > −8.0 K and can be clearly detected using multifocus imaging (see Fig. S2 and Supplementary Video). As reported in Fig. 1b, images taken at various depths Δ*z* near the sample surface show a characteristic interlaced fabric of ferroelectric domains. A 3D illustration of the observed section of the woven fabric is shown in Fig. 1b (left inset), composed of interlaced threads that weave “over" and “under" crossings with each other. The woven fabric is characterized by a distribution of multiply-linked threads with finite linking numbers (*LK*s in the language of knot theory), that describes how each node is woven, *LK* = + 1 for the over and *LK* = −1 for the under crossings^25^. Emerging during ferroelectric symmetry breaking, the spontaneous polarization woven fabric manifests a frozen history-dependent disorder, examples of which are shown in Fig. 1c, d. In detail, unlike a regular pattern, the spontaneously woven domain network shows a threading that depends randomly on the specific crossing point, with a random *LK* distribution (Fig. 1c). What’s more, the complexity and irregularity also occurs in the z direction, as revealed by close inspection (see Fig. 1d and Supplementry Video), where the fabric is not only formed by simple over and under crossings, but also has more complex threading geometries. The spontaneous domain weaving is found to be triggered by temperature during slow cooling (*α* < 0.4 K min^−1^) below the paraelectric-ferroelectric phase transition (Fig. 1e). At *T* − *T*_*C*_ = −1.95 K, a lattice of domains starts to transfrom into the woven fabric state (*T* − *T*_*C*_ = −2.00 K), which persists during further cooling until *T* − *T*_*C*_ = −8.00 K. Further lowering the temperature then causes the dissolving of domain walls, leading to a state with lower density of defects. Experiments involving multiple thermal cycles show that the *LK* distributions vary between different cooldowns. This suggests that it has the ability to store information topologically, i.e., that its geometrical structure can be caused to rearrange into a different pattern that is once again stable on experimentally relevant time scales, depending on external stimuli.

### Optical manipulation

**Fig. 2:**
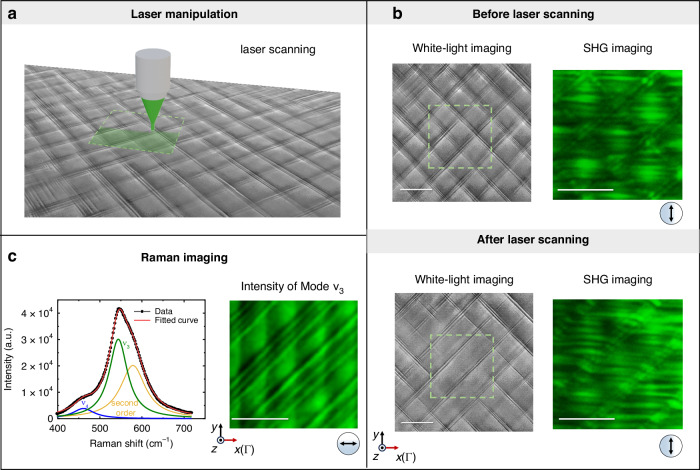
Laser-induced disentanglement of the domain fabric. **a** Representation of the focused-laser scanning scheme using confocal Raman setup with a 514 nm laser. **b** Top: Imaging of the woven domain fabric prior to 514 nm laser scanning using white-light microscopy (left) and confocal SHG microscopy (right). Bottom: Disentangled domain threads after laser scanning. **c** Left: Raman spectrum of the sample in the range of 400–730 cm^−1^, compared to the fitting result of Raman modes. Right: Intensity image of mode *ν*_3_. The polarization of the scanning laser used for Raman and SHG is along *x* and *y*, respectively. Scale bar is 10 μm

Laser manipulation of the woven fabric patterning is demonstrated using a confocal laser scanning system, as illustrated in Fig. 2a. A 514 nm laser is focused and scanned with a specific intensity and exposure time through the surface of the sample (see “Methods” section). Different complementary imaging techniques are used to detect the embedded domain distribution: white light imaging, second harmonic generation (SHG) microscopy, and confocal Raman microscopy. SHG microscopy is used to visualize domains and domain walls, that lead to characteristic constructive and destructive interference fringes in the converted SHG signal (see “Methods” section)^26–29^. Figure 2b displays the white light imaging and SHG microscopy of the woven domain fabric before (top panel) and after (bottom panel) the 514 nm laser scanning. The weaving pattern is observed to remain stable under the illumination of the 1040 nm femtosecond pulsed laser SHG pump. The green dashed region indicates the 514 nm laser scanning area, in which the originally interlaced domain structure (Fig. 2b top panel) is transformed into a disentangled layered structure (Fig. 2b bottom panel) by the light. Compared to the interlaced case, where the *LK* distribution contains non-trivial sequences that signal the woven fabric, the disentangled structure after laser illumination is left with only one direction of threads (those along 45 degrees in the case of Fig. 2b bottom panel). Which of the two possible diagonals is observed is random for each realization. In terms of topology, the transformation involves the “cutting" of the interlaced threads running in one direction (say, the “wefts”), leaving the second set of threads (i.e., the “warps”) untouched. The new locally disentangled pattern remains fixed until the temperature is appropriately changed, and the woven fabric can be restored by once again cycling the sample through *T*_*C*_, albeit in a different realization, that is, with a different *LK* distribution.

In addition to acting as a manipulation light, the 514 nm laser also serves as the excitation source for Raman scattering imaging. Owing to its sensitivity to lattice vibrations, Raman scattering provides a powerful means to examine polarization-related phenomena in perovskite oxides^30,31^. Specifically, the Raman mode *ν*_3_ (A_1*TO*_) near 550 cm^−1^ has been found to reflect the spontaneous polarization in tetragonal KTN^32^, with domain orientation and magnitude encoded in the mode’s spectral shape and shift. Domains oriented parallel to the laser polarization exhibit stronger and sharper mode intensity, while domains not aligned show weaker and broadened signals. The left panel of Fig. 2c displays a sample Raman spectrum in the range of 400–730 cm^−1^. According to density functional theory (DFT) calculations in ref. ^32^, the spectrum can be fitted by the combination of three different modes *ν*_3_ (A_1*TO*_), *ν*_4_ (E_*TO*_), and a second order mode. The intensity mapping of mode *ν*_3_ is shown in the right panel of Fig. 2c, where only disentangled parallel domain threads are observed, owing to the manipulation of the excitation laser itself.

### Modeling

**Fig. 3:**
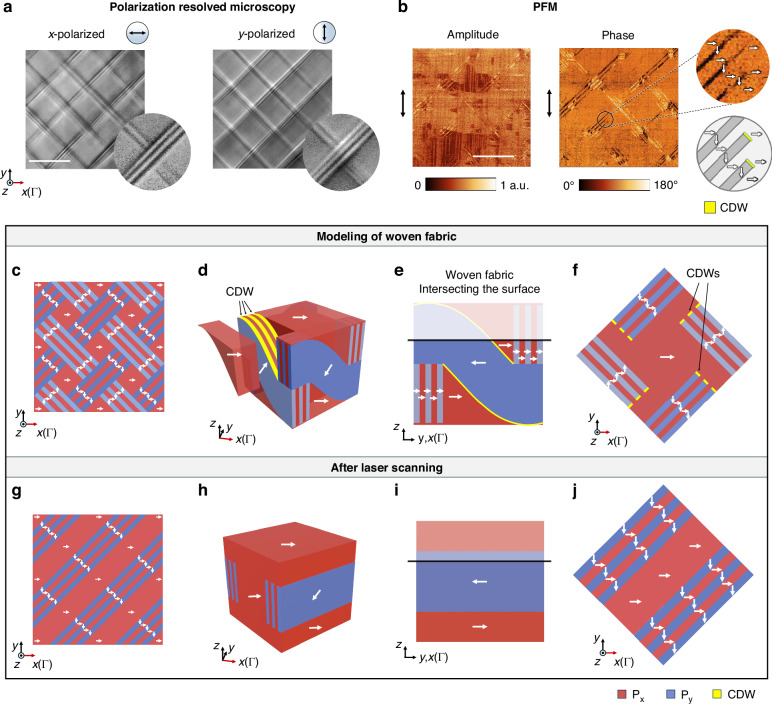
Modeling of woven domain fabric. **a** Microscopy of the woven fabric using (left) x- and (right) y-polarized white light. **b** PFM images of the domain structure at the sample surface and the evidence of the charged domain walls (CDWs). The amplitude and phase scans for a tip sensitivity along *y* (double arrows) with the enlarged sections (right) to illustrate the spontaneous polarization **P** (white arrows) and CDWs (yellow regions). **c**–**f** Modeling of woven fabric. **c** Inplane xy projection of polarization domains. **d** Full 3D unit cell with CDWs inbetween the prism shaped *P*_*x*_ domains and curved *P*_*y*_ fabric. **e** ($\overline{1}$10) plane side view of the woven fabric unit cell with CDWs. The black horizontal line represents the sample surface in the case that it intersects the woven fabric. The corresponding surface domain distribution is demonstrated in (**f**). **g**–**j** Same analysis for the more energetically stable unwoven fabric with no CDWs after laser scanning. Note that the white arrows indicate only possible polarization directions and do not preclude the existence of antiparallel domains within the same colored regions. Scale bar is 10 μm

Figure 3a shows the polarization-resolved microscope imaging of a woven fabric with x-polarized (left column) and y-polarized light (right column). Figure 3b displays details of the woven domains and spontaneous polarization distribution obtained at the sample surface using piezo-force-microscopy (PFM). The PFM amplitude and phase panels displayed have a direction of sensitivity to in-plane polarization **P** along the y-axis [**P** = (0, *P*_*y*_, 0)], with the growth direction of the sample Γ along the horizontal x-axis. The signal in the phase panel can be subdivided into three categories, bright, mid-tone, and dark, corresponding to positive, zero, and negative projections *P*_*y*_ of **P** along y, respectively. Thread-like domains are evident extending along the 45° and 135° diagonal directions, crossing each other. The mid-tone color regions in the phase panel correspond to prevalently *P*_*x*_ domains [i.e., with **P** = (*P*_*x*_, 0, 0)], due to the lack of out-of-plane *P*_*z*_^33^, as corroborated by phase contrast experiments in Fig. S3b and a full set of PFM images in Fig. S4. This is in agreement with the amplitude signal, where the thread-like regions that contain *P*_*y*_ domains have a correspondingly stronger signal than the surroundings. PFM results of spontaneous woven fabric domains forming at different depth in the crystal are reported in Fig. S5, consistent with the modeling in Fig. 1b. The smooth and regular topography of the PFM scan (see Figs. S4 and S5) indicates that for all the weaving, the sample remains a hard crystal and does not undergo any detectable deformation. Importantly, the surface scan reveals the expected disappearance of threads along one direction at the crossing points, consistent with weaving threads extending deeper into the bulk. In detail, as indicated in the blow-up PFM scan (Fig. 3b, right panels), domain threads are observed to terminate, making flux-closure impossible, forming CDWs at these ends, where tail-to-tail configurations develop (see the yellow shaded segments in the illustration panel). This is further corroborated by PFM scan of different sections of the weave, as reported in Fig. S5.

Figure 3c–f illustrates the corresponding modeling of woven fabric based on the formation of tetragonal polarization *P*_*x*_, *P*_*y*_, and *P*_*z*_ domains (see Fig. S3b), and their interaction with the constraint of reducing volume polarization charge. Phase contrast microscopy and PFM images indicate that *P*_*x*_ and *P*_*y*_ are the majority domains (see Fig. S3a), and the interwoven fabric appears dominated by so-called “superdomains” composed of periodic stripe domain bundles^34^. Fig. 3c shows the top view of woven domain modeling. As the domain pattern is observed to weave, domain walls incompatible with conventional 180° or 90° closed-flux configurations are present^35^, suggesting that they are CDWs. The model of a full 3D unit cell in Fig. 3d demonstrates CDWs inbetween the prism shaped *P*_*x*_ domains and curved *P*_*y*_ fabric. A ($\overline{1}$10) plane side view in Fig. 3e gives a clear picture of how the head to head and tail to tail domain walls form in 3D by weaving^36–40^. The CDWs emerge as locked into the woven fabric, with threads bending continuously and smoothly, in agreement with observations (see, for example, Figs. 1 and 3a, b). For the PFM experiments, we can only detect the woven fabric structures when they intersect the sample surface (depicted by the black horizontal line in Fig. 3e). The corresponding surface domain distribution is demonstrated in Fig. 3f with CDW distribution compatible with the PFM results shown in Fig. 3b bottom right.

Figure 3g–j shows the more energetically stable unwoven fabric after laser scanning, in which CDWs have been eliminated. This disentangling effect reflects a photoinduced rearrangement of local domain patterns into lower-energy configurations. It follows that the higher energy configuration associated with the CDWs is stablized by the topology of woven fabric during its spontaneous formation. Note that although the background regions (red *P*_*x*_) appear homogeneous under white-light imaging, SHG microscopy reveals horizontal 180° domain walls separating antiparallel *P*_*x*_ domains (*P*_*x*_ = ±*P*_*s*_). Similar arrangements are observed in the superdomains of the fabric meeting the constraint of minimizing volume polarization charge. Consequently, the white arrows in Fig. 3c–j indicate only possible polarization directions, i.e., both *P*_*x*_ = *P*_*s*_ and *P*_*x*_ = − *P*_*s*_ can exist within the same colored region, provided the arrangement satisfies the minimization of volume polarization charge constraint demostrated in Fig. 3c, g.

**Fig. 4:**
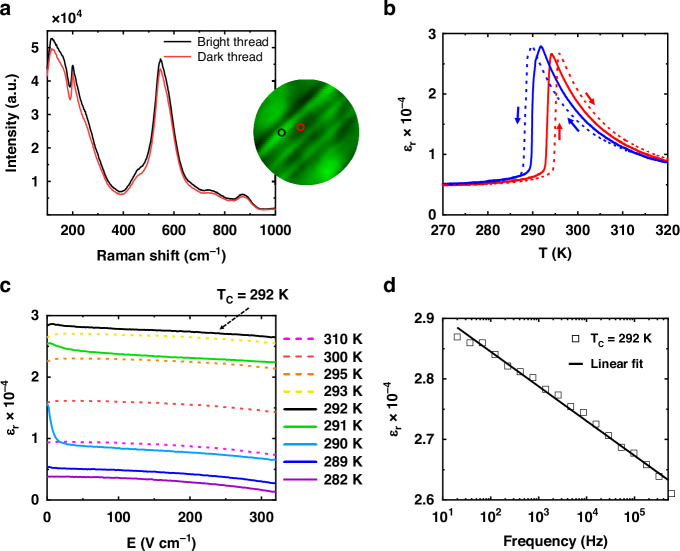
Raman spectrum and dielectric analysis. **a** Raman spectra of two points taken from the bright (black) and dark (red) threads of the woven fabric domains. **b**–**d** Non-ergodic features of the KTN:Li sample hosting the fabric state. **b** Thermal hysterisis loops of the low-frequency dielectric constant for different heating and cooling rates (solid lines for 3 K min^−1^, and dashed lines for 10 K min^−1^). **c** Low-frequency dielectric constant for different electric bias fields at different temperatures. **d** Dielectric constant dispersion near *T*_*C*_

In Fig. 4a, we report the full Raman spectrum relative to the Raman peak intensity distribution of the optically disentangled region, as in Fig. 2c, comparing regions associated to the bright and dark threads. The weakening of the Raman peaks for *P*_*y*_ is evident compared to those of *P*_*x*_. Fabric rearrangement caused by external stimuli signals a non-ergodic state. This is corroborated by the fact that the interlaced fabric forms only when the KTN:Li sample is slowly cooled (*α* < 0.4 K min^−1^) through the Curie point to its final equilibration temperature (*T*_*C*_ − 2 K > *T* > *T*_*C*_ − 8 K). Evidence of macroscopic thermal hysteresis and non-ergodic response to electrical signals near *T*_*C*_ is reported in Fig. 4b–d. The low frequency (*ω* = 130 Hz) relative dielectric constant *ε*_*r*_*versusT* for different cooling rates shows a significant thermal hysteresis during the phase transition (Fig. 4b). Furthermore, for a given *T*, the values of low-frequency *ε*_*r*_*versus* bias electric field *E*, reported in Fig. 4c, shows a strong susceptibility for *T* = *T*_*C*_ − 2 K = 290 K, at the temperature for which weaving is observed to set in as a sudden transition. High frequency spectroscopy of KTN:Li at different *T* and estimates of the optical index of refraction can be found in ref. ^41^. In Fig. 4d, we report the low frequency dielectric response, which shows finite dispersion at *T*_*C*_ = 292 K, a feature that typically accompanies glassy behavior in relaxor-like ferroelectrics that, in our case, may be caused by random electric fields associated to compositional disorder in the Nb/Ta atoms^42^. Further physical insight into the domain pattern formation mechanism and how it relates to frustration and nonergodicity can potentially be provided by energy landscape modeling using theoretical calculations^7,21^.

## Discussion

The spontaneous formation of the domain fabric reported in Fig. 1 hosts embedded, topologically stabilized CDWs, as illustrated in Fig. 3b–f. Illumination of the fabric using intense focused visible laser light (514 nm) causes the fabric to transform into an unlinked cross-hair like structure, as described in Fig. 2. An analogous disentangling effect is not observed using longer near infrared wavelengths (1040 nm). Since weaving is only observed on slow cooling in a specific temperature range, the fabric can be associated to a local minimum in the free energy of the system. In this vein, optical disentanglement represents a transition to a lower free energy state, where the electrostatic energy associated to the CDWs is absent. Furthermore, the band gap of the material is located near 400 nm^41^, so that the observed wavelength dependence is unlikely to originate from differences in intrinsic lattice absorption^43^, and no amorphization or irreversible structural damage is observed^44^. A candidate explanation for the disentangling effect is that it is driven by photoinduced charges from deep in-band impurities, a well-known mechanism associated with photorefraction in KTN:Li (see, for example, ^45^). The photoinduced carriers can then screen the bound charges associated with CDWs, thereby reducing their electrostatic energy and enabling relaxation towards a more energetically favorable polarization distribution. This picture is corroborated by the response of the fabric to a bias electric field. Specifically, we found that a field above 200 V mm^−1^ begins disentangling the domains as shown in Fig. S6a. The threshold (coercive) field is identified through polarization-versus-electric-field (P-E) and current-versus-electric-field (I-E) loop experiments (see Figs. S6b,c), where a well-localized peak in the current at approximately 250 V mm^−1^ is found, a field that is smaller than the photorefractive saturation field, i.e., it can be generated by photoinduced space charge^45,46^. We note that domain susceptibility may also be caused by increased local internal polarizability of both domains and domain walls^47^. That photorefraction is at play is further corroborated by the fact that the disentangling phenomenon is cumulative in time, as typical for photorefractive space-charge build-up.

Domain weaving is introduced to describe the interlaced features observed using 3D imaging. The result is a non-trivial 3D structure that is in sharp contrast to previously reported domain maps, that are generally 2D^48–50^. Compared with previously studied topological defects in ferroelectrics, such as domain walls, vortices, and skyrmions^5–8^, which are typically localized and weakly connected, the woven structure is formed by entangled threads, giving rise to delocalized global topological protection^13,14,25^. The woven structure then introduces a new approach to conceive optically addressable ferroelectric memory based on interlaced global topological protection, where different metastable woven states can emerge in the same experimental conditions, each state rendered accessible by the previous thermal history along with the details of the optical exposure. While ferroelectric superlattices can and have been engineered, engineering a woven fabric would involve the basic challenge of having to thread domains together. In our experiment, this hurdle is overcome by spontaneous symmetry breaking that naturally generates the woven infrastructure, which then has a laser-activated form of topological flexibility.

In summary, we have reported the direct observation of an interwoven network composed of ferroelectric domains spontaneously formed in KTN:Li, a hard transparent crystal. Pooling together multifocus optical imaging, PFM, Confocal Raman Spectroscopy, SHG imaging, and phase-contrast experiments, we propose a model based on the constraint of reducing volume polarization charge to rationalize how the temperature-driven weaving emerges. Protected by the woven topology, the structure supports CDWs at the interfaces of curved domains, that can be optically driven by a focused 514 nm visible laser into a lower-energy, disentangled state. The woven architecture remains robust within the temperature range *T*_*C*_ − 2K > *T* > *T*_*C*_ − 8K, and preserves its stability under 1040 nm infrared scanning. Our findings suggest the possibility of developing a wholly innovative technological platform. For example, the interwoven fabric hosted in solid-state could be used for topologically protected photonic memory and energy storage, that, as shown in our experiments, can be optically detected and manipulated, a new state of 3D polar systems that can prove instrumental in the development of innovative computing ideas, such as in optical processing and neuromorphic computing^3,51–53^. What’s more, as a product of spontaneous-symmetry-breaking, this new type of global topological defect is expected to be of a universal nature, occurring in other topology-driven systems, such as liquid-crystals, superconductors, quantum fields, and the cosmology of the early universe.

## Materials and methods

### Materials

The KTN:Li crystal is grown using the top-seeded solution method, a zero-cut 3.4 mm (x) × 2.1 mm (y) × 0.5 mm (z) extraction as shown in Fig. S1a. The optical quality sample has an average composition K_0.997_Ta_0.62_Nb_0.38_O_3_:Li_0.003_, with an average Curie temperature *T*_*C*_ = 292 K. As the sample is cooled through *T*_*C*_, the perovskite lattice experiences a structural transition from a cubic to a tetragonal phase accompanied by the formation of domains. The pulling growth procedure introduces a residual built-in oscillation in the composition that leads to an approximately periodic striation pattern along the growth axis Γ (the *x* axis), as described in ref. ^24^. Figure S1b (top panel) reports the microscope image taken near *T*_*C*_, using y-polarized light, revealing the periodic striation patterning of the crystal. The slight variation in the substitutional disorder of Ta^5+^ ions with Nb^5+^ ions and K^+^ ions with Li^+^ influences the phase transition, resulting in a periodic distribution of *T*_*C*_(*x*) around average *T*_*C*_ illustrated in Fig. S1b (bottom panel). The striation period can be tuned either by changing the crystal rotational speed during growth or by blowing air into the chamber periodically. In this manner, we have been able to change the built-in striation lattice constant from 0.5 to 10 μm. During the phase transition, this periodicity along the growth direction is further transferred to a second, and ultimately even a third crystal axis by the slanted 90° domain walls, compatible with the polar tetragonal lattice symmetry^15,16^. As regards to sample thickness, we have observed woven domains in samples from 24 to 500 μm. In thicker samples, fully developed 3D domain patterns are found^15,16,20,21^.

### White-light optical microscope and 3D imaging

For the observation of woven domain fabric, the sample is cooled through the Curie point to *T*_*C*_ − 2.0 K > *T* > *T*_*C*_ −8.0 K with a slow cooling rate of *α* < 0.4 K min^−1^. Faster cooling to *T* − *T*_*C*_ = −2.0 K results in the transformation of the domain lattice into an irregular state similar to Fig. 1e last column (*T* − *T*_*C*_ = −9.0 K), with no appearance of woven domain fabric. The woven fabric is fully reproducible by once again cycling the sample through *T*_*C*_, albeit in a different realization, that is, with a different *LK* distribution. A given weave is observed to remain unaltered at a fixed temperature, with aging experiments conducted for up to 6 h. Real-space white-light imaging of woven domain fabric and ferroelectric domain evolution is obtained using an Olympus BX43 microscope with a 50 × (NA = 0.80) objective and a 100 × (NA = 0.90) objective. The resolution and the depth of field of the system is approximately 0.9 and 3.0 μm for the 50 × (NA = 0.80) objective, and 0.8 μm and 2.1 μm for the 100 × (NA=0.90) objective, respectively. The sample is mounted on a piezo stage, which can scan along the *z* axis (Fig. S2) to reconstruct the 3D images of the woven structure in Fig. 1b. White light experiments are performed with linearly polarized light along either the *x* (growth) or *y* directions. Optical contrast originates from birefringence, and is caused by differences in refraction, reflection, and scattering in the different domains. In the figures, when not specified, light is polarized along the x (growth) direction. Images are representative cut-outs of larger images.

### Phase-contrast microscopy

Phase-contrast images are taken using the Olympus BX51 microscope system with an “UPlanFL N 100x” immersion objective with phase-contrast. Polarization-resolved images are recorded for different linear polarization states. Sample angles between the optical polarization and the growth direction Γ (x-axis), namely 0°, 45°, 90°, and 135°, are reported in Fig. S3a. Analyzing the index of refraction distribution versus orientation of the optical polarization used allows us to identify the birefringence and hence spontaneous polarization **P** distribution of the domains in the xy-plane. Fig. S3a reports optical polarization-resolved phase-contrast imaging of the formation stage of the woven fabric at *T* − *T*_*C*_ = −1.95 K, where the alternating in-plane *P*_*x*_ and *P*_*y*_ striped pattern (pink dashed regions) emerges. The light polarization denpendent behavior indicates that the in-plane *P*_*x*_ and *P*_*y*_ domains are dominant polarization of the woven fabric. What’s more, the fact that the 45° and 135° images are the same in Fig. S3a indicates that the in-plane projection of the spontaneous polarization is not along the diagonals for woven domain fabric state, as compatible with the cubic-to-tetragonal phase transition (Fig. S3b).

### Piezo-force microscopy

We perform piezoforce microscopy (PFM) with an atomic force microscope (AFM, Bruker) for simultaneous acquisition of topography, in-plane, and out-of-plane PFM responses (Fig. S4). All PFM measurements are carried out on the (001)-oriented crystal face (the xy plane in our reference system), while silver paste was painted on the opposite face to serve as the bottom electrode. During the experiment, the position of the woven fabric is irregular in the *z* direction, forming at a random history-dependent depth near the sample surface. Hence, while the optical microscope image of the fabric is always detectable, its PFM signal is only observed in the subset of realizations where it extends and intersects the surface, as reported in Figs. 3b, S4, and S5. Note that PFM scans also show position-dependent random-like surface structures that are not observed in optical imaging. These are present also when the fabric is absent, and are sometimes absent in the lateral PFM phase images. Different scans in different positions indicate that they do not affect the woven fabric, meaning that the PFM signal is a superposition of the woven fabric on a surface structure background, see Fig. S5.

### Dielectric measurements

Dielectric measurements as a function of temperature reported in Fig. 4b are performed applying a low voltage (1 V) low frequency (130 Hz) AC field to the crystal along the *z* axis using electrodes deposited on the top and bottom of the (001)-oriented crystal face. Fig. 4b shows thermal hysteresis during the heating and cooling process at different rates 3 K min^−1^ and 10 K min^−1^. Figure 4c shows dielectric measurements at different temperatures with a DC bias field (changing from 0 to 350 V cm^−1^). For these, the crystal is first heated to 333 K, and then cooled at 0.5 K min^−1^ to the specific working temperature, whereupon we perform our measurements. Dielectric spectroscopy reported in Fig. 4d is performed with the 1 V low voltage AC field at different frequencies from 20 Hz to 600 KHz.

### Laser manipulation and Raman imaging

Laser manipulation is performed using a Renishaw confocal system, which is designed for Raman imaging and offers a spectral resolution better than 1.5 cm^−1^. A 514 nm laser is focused using an Olympus 50 × objective (NA = 0.80) down to a diameter of *D* = 0.8 μm, with an average intensity of *I* = 6.3 × 10^6^ W cm^−2^. To achieve the disentanglement, the sample is scanned by the laser in the xy-plane for an exposure time of 1 s at each step, with the step distance of 0.5 μm in a 20 × 20 *μ*m region. At each step, the Raman spectrum is collected for imaging analysis.

### Second harmonic generation microscopy

Second-harmonic generation (SHG) imaging was performed in a laser-scanning microscopy configuration using a 1040 nm femtosecond laser with an average power of 100 mW, a pulse duration of 79 fs, and a repetition rate of 34 MHz. The excitation beam was focused onto the sample using an Olympus 40 × objective with a numerical aperture of 0.95, and the SHG signal was collected during raster scanning of the region of interest. The incident fundamental light was linearly polarized along the in-plane y direction. The emitted SHG signal was collected without polarization analysis. In the tetragonal ferroelectric phase, KTN belongs to the 4*mm* point group. The second-order nonlinear polarization can be written as

$${P}_{i}^{(2\omega )}={\varepsilon }_{0}\mathop{\sum }\limits_{jk}{\chi }_{ijk}^{(2)}{E}_{j}^{(\omega )}{E}_{k}^{(\omega )}$$

or, equivalently, using the contracted *d*-tensor notation,

$$\left(\begin{array}{c}{P}_{x}\\ {P}_{y}\\ {P}_{z}\end{array}\right)=\left(\begin{array}{cccccc}0 & 0 & 0 & 0 & {d}_{15} & 0\\ 0 & 0 & 0 & {d}_{15} & 0 & 0\\ {d}_{31} & {d}_{31} & {d}_{33} & 0 & 0 & 0\end{array}\right)\left(\begin{array}{c}{E}_{x}^{2}\\ {E}_{y}^{2}\\ {E}_{z}^{2}\\ 2{E}_{y}{E}_{z}\\ 2{E}_{x}{E}_{z}\\ 2{E}_{x}{E}_{y}\end{array}\right)$$

where the nonvanishing second-order nonlinear coefficients are *d*_15_ = *d*_24_, *d*_31_ = *d*_32_, and *d*_33_. The SHG response is therefore directly sensitive to the spontaneous polarization and provides a direct probe of ferroelectric domain configurations.

## Supplementary information

Supplemental material in support of main text

Multifocus imaging of the fabric

## Data Availability

All data needed to evaluate the conclusions in the paper are present in the paper. Additional data related to this paper may be requested from the authors.
