# Supplementary material for: Spontaneous formation and optical manipulation of a woven domain fabric in a ferroelectric crystal
Source: Light Sci Appl. 2026 Jul 14;15:315. doi: 10.1038/s41377-026-02374-7 (PMC13370020; doi:10.1038/s41377-026-02374-7)
Supplement: Supplementary file 1 — Supplemental material in support of main text [file 41377_2026_2374_MOESM1_ESM.pdf]

Supplemental Material

Spontaneous formation and optical manipulation of a woven domain fabric in a ferroelectric crystal

Feifei Xin,[1,2] Yehonatan Gelkop,[3] Ewout van der Veer,[4] Beatriz Noheda,[4,5] Ludovica Falsi,[2] Guoquan Zhang,[1] Fang Bo,[1] Aharon J. Agranat,[3] and Eugenio DelRe[2,*]

[1] *The MOE Key Laboratory of Weak-Light Nonlinear Photonics, School of Physics and TEDA Applied Physics Institute, Nankai University, Tianjin 300071, China.*
[2] *Dipartimento di Fisica, Università di Roma "La Sapienza", Rome 00185, Italy.*
[3] *The Institute of Applied Physics, The Hebrew University, Jerusalem 91904, Israel.*
[4] *Zernike Institute for Advanced Materials, University of Groningen, Groningen 9747AG, The Netherlands.*
[5] *Groningen Cognitive Systems and Materials Center (CogniGron), University of Groningen, Groningen, 9747AG, The Netherlands.*

(Dated: May 21, 2026)

* contact author: eugenio.delre@uniroma1.it

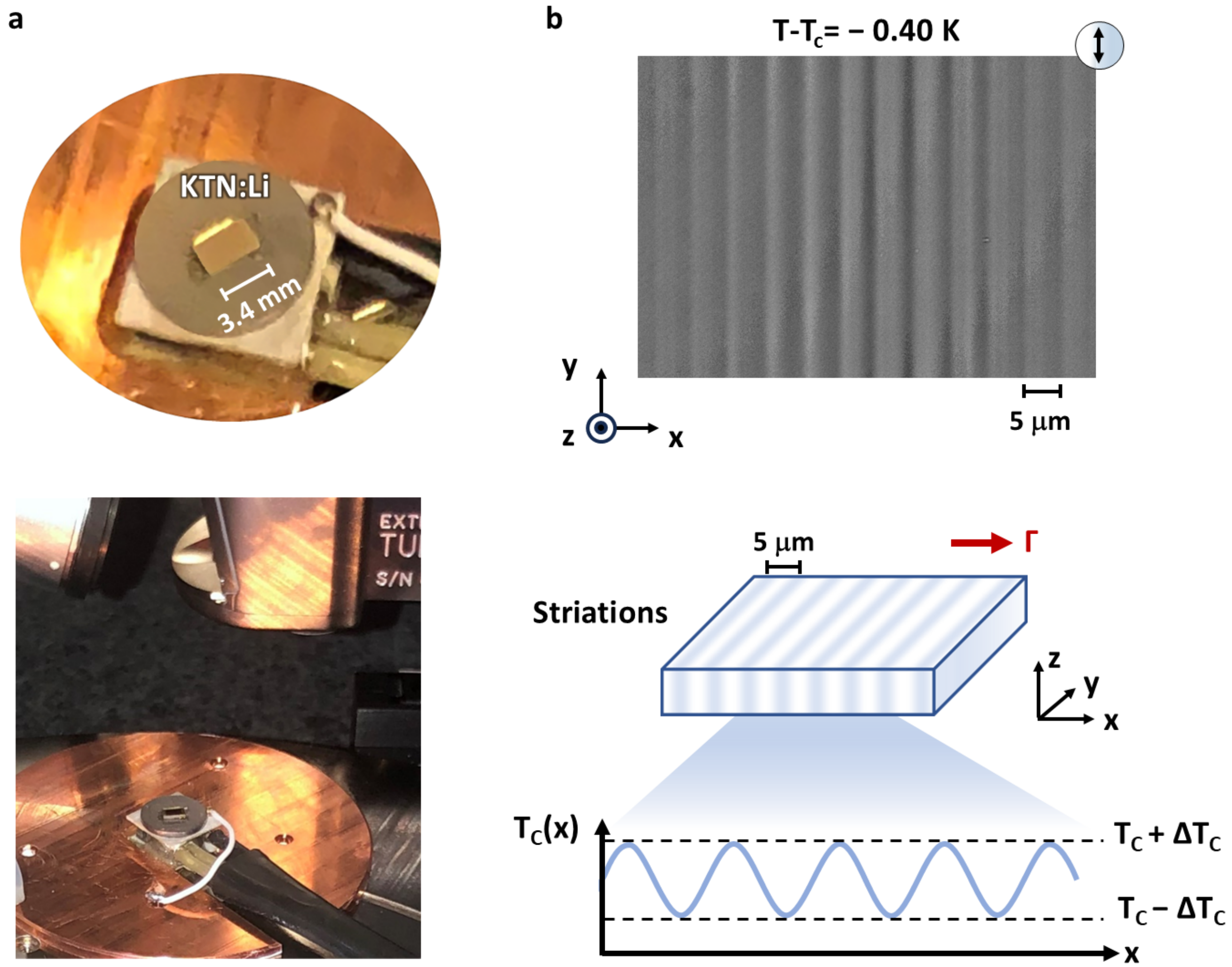

FIG. S1: **KTN:Li sample and striations. a** Snapshots of the sample on the temperature control stage. **b** Top: microscope image taken near T_C using y-polarized light. Bottom: illustration of the periodic distribution of $T_C(x)$ along the growth direction Γ (i.e., x-axis) of the sample associated to the built-in striation pattern with a period of 5 μm.

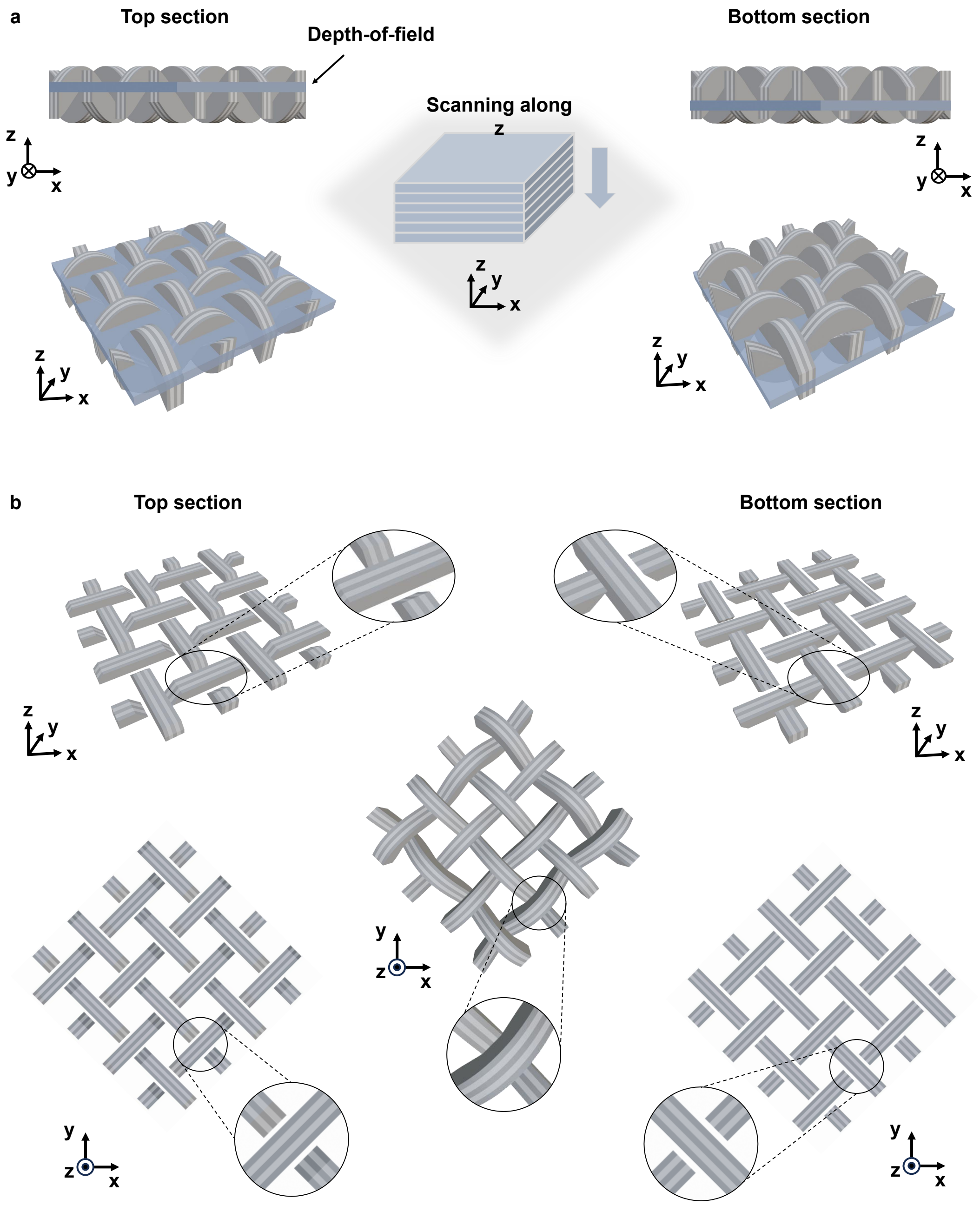

FIG. S2: **Illustrating scanning along z. a** The x and y axes are the principal in-plane (sample surface) crystal axes and z is the out-of-plane axis. The 3D images of woven fabric are reconstructed by scanning along the z-axis. As the microscope objective is translated in the z direction (central panel), the finite depth-of-field means that only a section of the pattern (illustrated in blue shade) is imaged, for example, the top section (left panels) and bottom section (right panels). **b** The result of this sectioning of the interlaced structure (central panel) leads to two different images, left panels for the top section, and right panels for the bottom section.

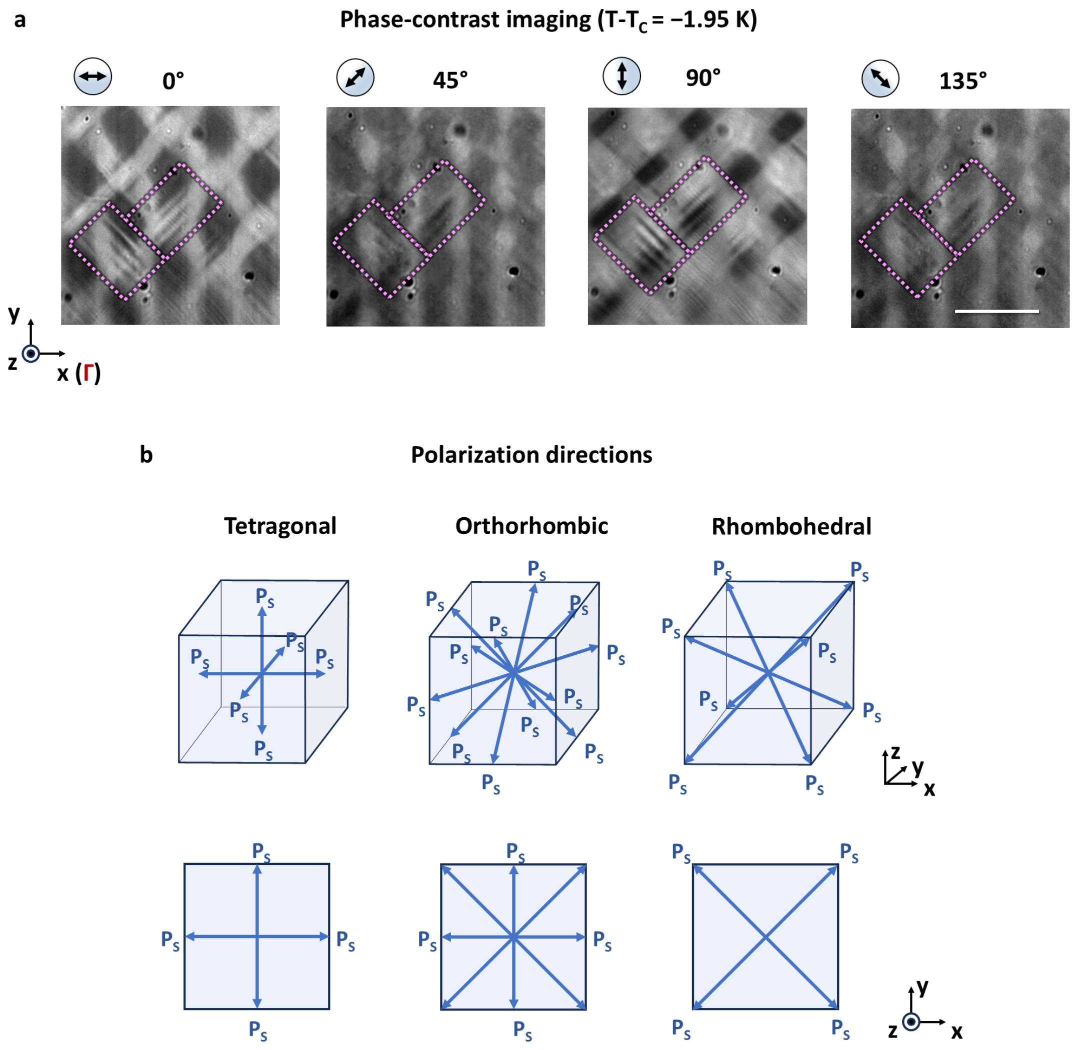

FIG. S3: **Phase-contrast imaging and ferroelectric phases.** **a** Phase-contrast imaging at $T - T_C = -1.95$ K during the formation of the woven fabric state, using different optical polarizations (double black arrows). Emerging striped regions with alternating in-plane P_x and P_y polarized domains are flagged (pink rectangles). Note that the rhombus-like regions are also sensitive to the polarization of the probe light, indicating that the in plane P_x and P_y domains are dominant in the woven fabric state. Scale bar is 10 μm. **b** 3D illustration (top) and x-y projection (bottom) of allowed spontaneous polarization directions for the tetragonal, orthorhombic, and rhombohedral perovskite phases.

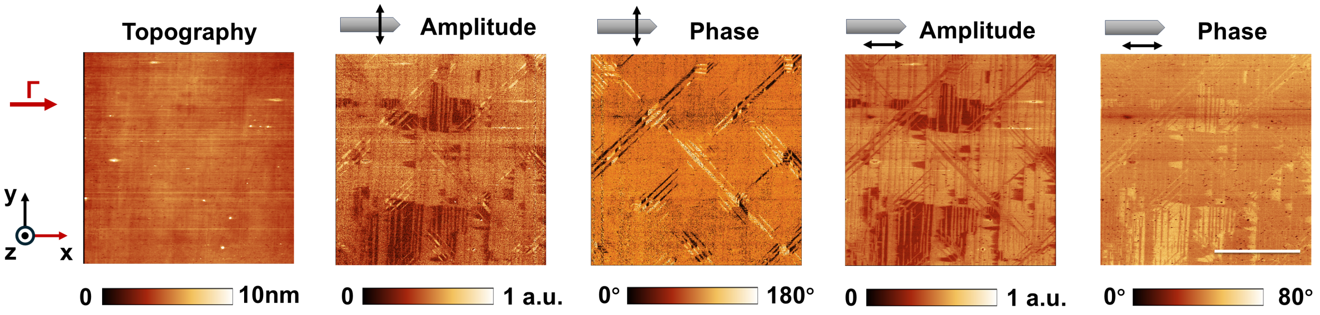

FIG. S4: **PFM images of woven domain fabric at the sample surface.** Topography, lateral amplitude, lateral phase, vertical amplitude, and vertical phase PFM images are reported from left to right. The growth direction of the sample is along the horizontal x direction. The cantilever orientation is indicated by the solid grey pointer. Considering the absence of P_z corroborated by the phase-contrast images in Fig. S3a, apart from the irregular surface defects of the sample, the weak patterning for the out-of-plane vertical PFM phase can be attributed to the cantilever buckling effect in the presence of in-plane P_x [33]. The direction of sensitivity to the spontaneous polarization is indicated by double black arrows. Extraoneous patterns observed, for example in the amplitude panels, are crystal position-dependent defects that are not observed in the optical imaging, suggesting that they are associated to surface inhomogeneities and not to the spontaneous ferroelectric pattern formation process. Scale bar is 10 μm.

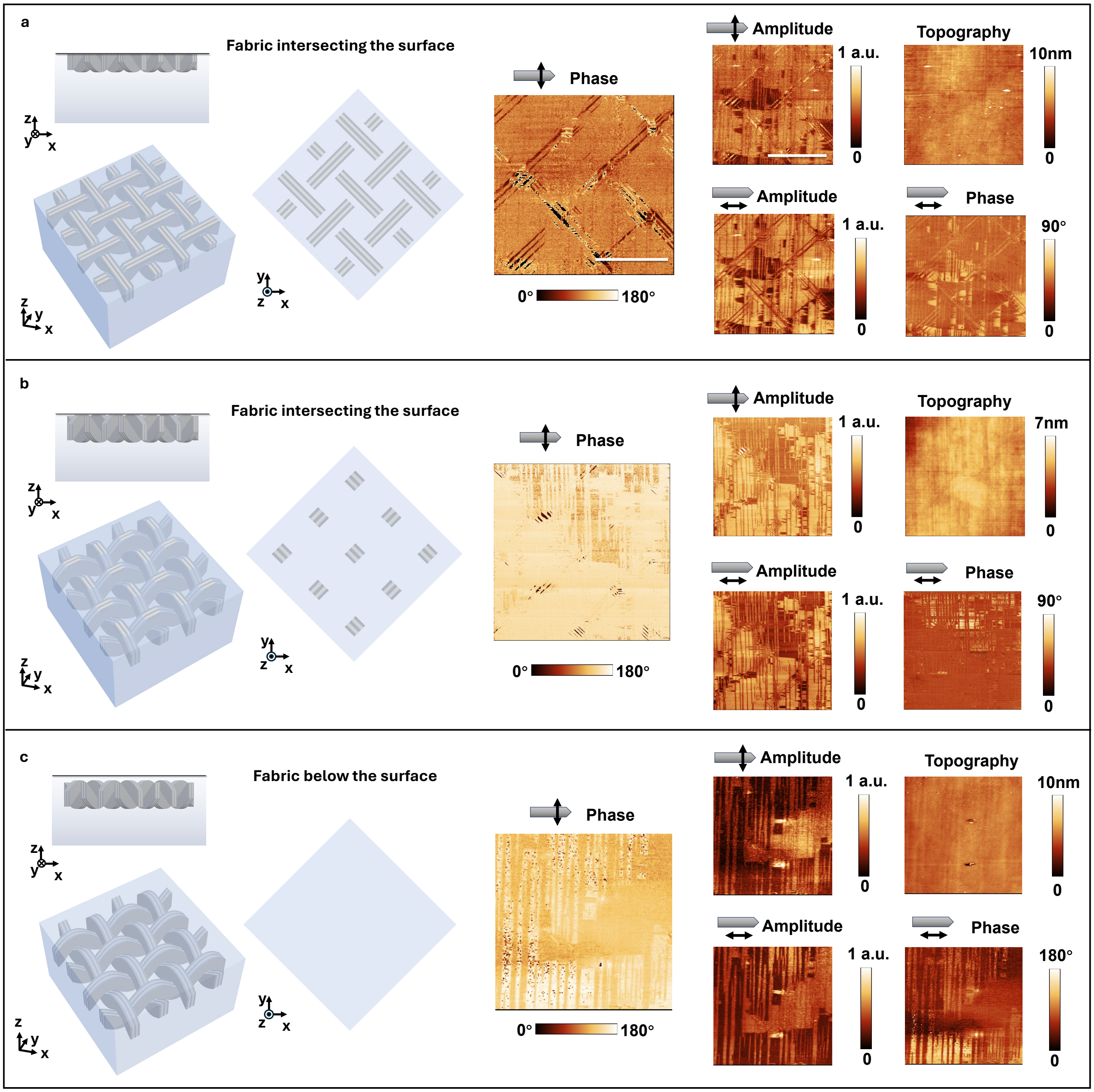

FIG. S5: **PFM images of woven fabric structure at different depth.** **a** Left panel: modeling of woven fabric intersecting the sample surface. Almost half of the upper part is "cut off", revealing a clear domain structure of the weaving at the sample surface. Right panel: corresponding PFM images of the woven domain fabric. **b** Left panel: modeling of woven fabric at a lower position but still intersecting the sample surface. Only a small portion of the upper part is "cut off", revealing the crossings of the woven domain fabric at the sample surface. Right panel: corresponding PFM images of the crossings of the 3D domain structure. **c** Left panel: modeling of woven fabric below the sample surface. Right panel: corresponding PFM images showing no woven fabric, even though we can observe the woven fabric using optical microscope. Scale bar is 10 μm.

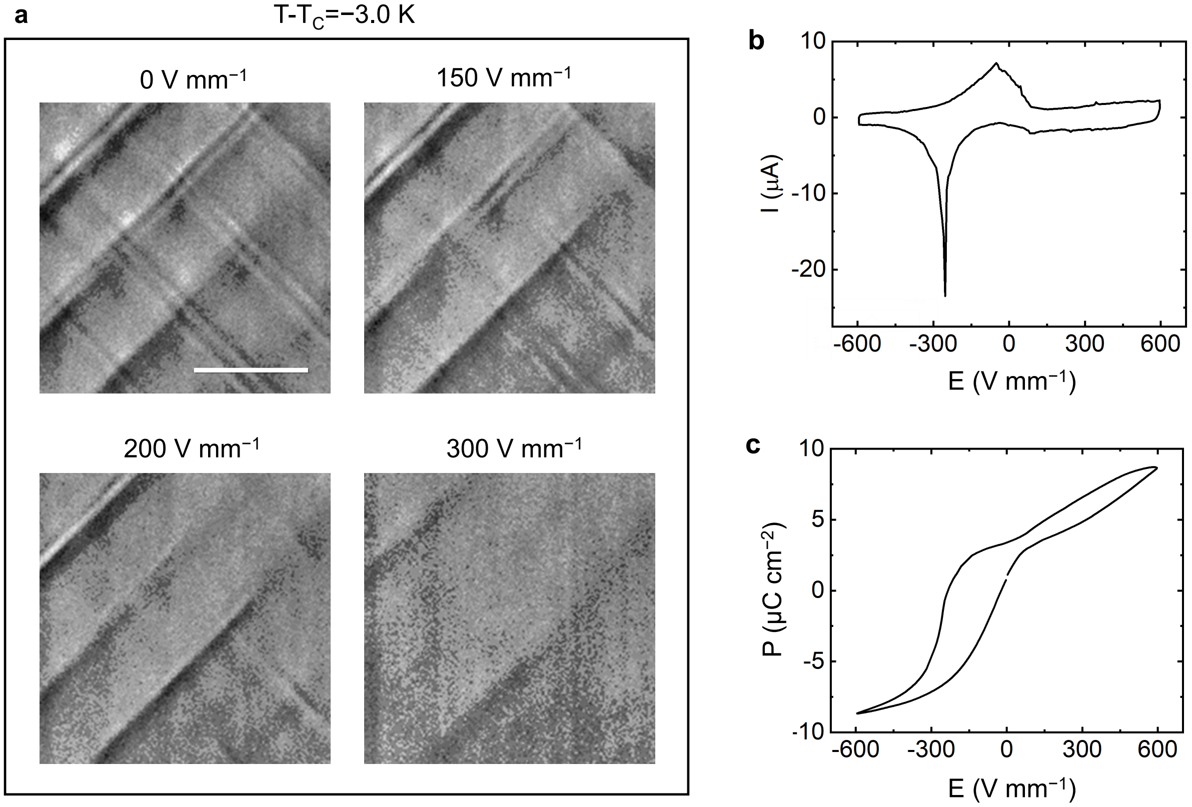

FIG. S6: **Woven fabric and electric field bias.** **a** Images of the woven domain pattern at $T - T_C = -3.0$ K for increasing values of electric bias field E. **b** Current intensity I versus E and **c** polarization P versus E loop plots in conditions in which the fabric forms. The localized peak at $E = -250$V mm^{-1} indicates the threshold field for domain structure breakdown, while the asymmetry indicates the presence of an internal field that, for temperatures close to T_C where the fabric forms, naturally leads to strongly distorted loops [46]. Scale bar is 10 μm.
